# Task-Sharing and Stepped-Care Mental Health Services in Angolan Higher Education: A Multi-Site Implementation Protocol (ANGOSAP)

**DOI:** 10.12688/openreseurope.23442.2

**Published:** 2026-06-17

**Authors:** Alvaro Rodríguez-Mora, Jose I. Navarro, Mariló Gómez-Domínguez, Nina Czarnecka, Inidia Rubio, Sandra Mariza M. da Cruz, Peter Engert, Claudia Preißer, Jerónimo S. Mendes Evaristo, VilmaTeresa Dos Santos José, Jean Marie Kiombo, Mbaz Nauege, Evaristo Vitangui Gando, Henriques Jamba Dachala

**Affiliations:** 1 Universidad de Cadiz Departamento de Psicologia, Puerto Real, Cádiz, 11519, Spain; 2 Oficina de Relaciones Internacionales, Universidad de Cadiz, Cádiz, 11001, Spain; 3 Universidade Oscar Ribas, Luanda, Luanda Province, Angola; 4 RheinMain University of Applied Sciences, Wiesbaden, Hesse, 65022, Germany; 5 Universidade do Namibe, Namibe, Namibe Province, Angola; 6 Universidade Lueji A’Nkonde, Dundo, Angola; 7 Instituto Superior de Ciências da Educação do Bié, Bié, Angola

**Keywords:** Angola; university students; mental health services; psychoeducation; stepped care; capacity building; task sharing; implementation; Sustainable Development Goals.

## Abstract

**Background:**

Higher education institutions in Angola face increasing demand for mental health and psychoeducational support in the context of limited specialist workforce and service capacity. ANGOSAP is an Erasmus+ − funded capacity-building initiative designed to establish sustainable, university-embedded Psychological and Psychoeducational Support Services across partner institutions in Angola and to strengthen early access to support within higher education settings.

**Methods:**

This Study Protocol describes the rationale, service model, and early implementation framework of ANGOSAP. The project uses a co-designed, stepped-care, task-sharing approach adapted to the context of each participating institution. Core components include campus-level standard operating procedures for intake, triage, risk assessment, safeguarding, and referral; competency-based training and supervision; student-facing psychoeducation, brief psychological interventions, and digital resources; formal referral pathways; and pragmatic monitoring. Early implementation success will be assessed using predefined indicators: service reach, median waiting time from first contact to first appointment, first-appointment uptake, care-plan completion, reliable clinical change on validated symptom or functioning measures, serious incidents, acceptability, and equity-stratified access and outcomes.

**Results:**

No primary clinical outcomes or human participant data are reported in this Study Protocol. Preliminary outputs include a defined implementation pathway for university-based services, a minimum dataset for routine monitoring, quality and safety procedures, equity-oriented performance dashboards, and a cross-site learning structure to support iterative service refinement. These elements are intended to enable early-access, context-sensitive care while generating practice-based evidence during phased implementation.

**Conclusions:**

ANGOSAP provides a structured protocol to test the feasibility of university-based psychological and psychoeducational support in Angola. By combining capacity building, supervision, referral integration, and routine monitoring, the project will generate practice-based evidence on reach, access, safety, acceptability, and early clinical change. These findings will inform service refinement across participating institutions and provide a roadmap for engaging national stakeholders, including health and higher education authorities, in future policy development.

## Plain Language summary

Angolan universities are seeing an increasing number of students who need help with stress, anxiety, depression, academic difficulties, and adjustment. However, trained specialists are few, and many campuses have no dedicated service. ANGOSAP is creating university-based Psychological and Psychoeducational Support Services that combine good information (psychoeducation), brief talking therapies, and clear pathways to specialized care when needed. The model trains existing staff (for example, counselors, psychologists, and student support officers) and provides them with supervision and tools so that students can receive help earlier and closer to where they study. We describe why this is needed in Angola, how the service will work, and how we will ensure that it helps students fairly and effectively. This manuscript is presented as a Study Protocol following Open Research Europe guidance.

## Introduction

Angola’s population is young and urbanizing, and its Higher Education Institutions (HEI) are central to social and economic development. Similar to many countries in sub-Saharan Africa, Angola faces a dual challenge: high and possibly rising demand for mental healthcare and a historically under-resourced system. This demand was further amplified by the coronavirus disease 2019 (COVID-19) pandemic, which increased the global prevalence and burden of depressive and anxiety disorders in 2020 ( Santomauro et al., 2021). Recent GBD 2021 analyses identified Angola among countries with the highest age-standardized incidence rate (ASIR) of mental disorders, consistent with regional patterns seen in central sub-Saharan Africa ( Fan et al., 2025; GBD 2021 Diseases and Injuries Collaborators, 2024). Meanwhile, the specialist mental health workforce in the public sector remains limited; the latest World Health Organization (WHO) country profile (2017) reported approximately 0.06 psychiatrists and 0.18 psychologists per 100,000 population, with approximately 1.49 total mental health workers per 100,000 ( World Health Organization, 2017).

Mortality due to suicide remains a key measurable outcome in mental health systems. The World Health Organization’s most recent global country-resolved baseline (2019) under the Global Health Estimates shows varied patterns in the African Region; for example, in Angola, World Bank/WHO-compiled indicators report a suicide mortality rate of approximately six per 100,000 in 2019, underscoring the need for prevention, early identification, and care pathways ( World Health Organization, 2021).

Against this backdrop, the ANGOSAP project ( https://angosap.org/en/) aims to establish sustainable, university-embedded Psychological and Psychoeducational Support Services that expand access using a stepped-care, task-sharing model and strengthen referral links with public specialist care where available. The present Study Protocol distills the project’s rationale, design, and early implementation framework from the internal technical description and grant proposal.

### Study aims and rationale

The overarching objective of ANGOSAP is to improve student well-being and learning outcomes by building campus-based services that provide (i) psychoeducation, (ii) screening and early intervention for common mental disorders and academic-related difficulties, (iii) crisis procedures and safeguarding, and (iv) structured referral to specialist care. In Study Protocol form, the manuscript sets out the rationale, operational model, and implementation procedures through which this capacity-building effort will be established and evaluated across partner higher education institutions in Angola. Three converging signals justify action:

1. Burden and unmet need. The Global Burden of Disease Study 2021 highlights high incidence rates in Angola within the region ( Fan et al., 2025).
2. System constraints. According to World Health Organization (WHO) country profile documents, there are very low specialist densities and limited dedicated outpatient capacity ( World Health Organization, 2017).
3. Policy momentum. World Health Organization Angola’s 2024 Annual Report describes ongoing health-system strengthening, including policy revision and information-system improvements, an enabling context for university-level service innovation that dovetails with national priorities ( World Health Organization, 2024).

Complementary evidence from the GBD 2019 age-period-cohort analyses highlights changing incidence patterns for depressive disorders over 1990–2019, supporting the need to strengthen early identification and brief interventions ( Wu et al., 2024).

### Study design and methodology

This Study Protocol describes a multi-site, co-designed, stepped-care, task-sharing implementation model for establishing university-embedded Psychological and Psychoeducational Support Services across participating higher education institutions in Angola. The protocol combines locally adapted service design, standardized operational procedures, workforce development, referral coordination, and a pragmatic evaluation framework intended to generate actionable implementation learning during early scale-up.

### Design principles

Building on the preceding rationale, the protocol is articulated through a set of core design principles, which are described individually below. Together, these principles define how the service is structured, implemented, and adapted to different institutional realities, ensuring coherence between the conceptual framework, operational procedures, and the needs of students and staff.

a) Co-design and context fit. Each participating HEI will complete a structured service-design workshop with administrators, student representatives, and clinicians to adapt the core service blueprint to local realities (language, staffing, timetables, and infrastructure).
b) Stepped care. Tier 1: mental health literacy and psychoeducation; Tier 2: brief, evidence-based psychological interventions (e.g., problem-solving therapy, behavioral activation, and anxiety management) delivered by trained specialists under supervision; Tier 3: referral to specialist care for severe or complex conditions.
c) Task sharing with supervision. Competency-based curricula and supervision ladders align with global practices in low-resource settings, prioritizing safety, fidelity, and scalability.
d) Equity and inclusion. Active outreach to first-generation students, women, and students with disabilities; confidential access points; and multilingual materials. Ethical practice will be reinforced through informed consent, confidentiality, and data protection procedures proportionate to local regulations and the sensitivity of students’ mental health information

### Core components

Building on the design principles outlined above, the protocol is operationalized through a set of core components that are described individually below. Together, these components specify how the service will be governed, staffed, delivered, and continuously improved, thereby linking the conceptual framework to day-to-day practice in a way that can be implemented consistently across participating institutions.

1. Governance & protocols. Campus-level Standard Operating Procedures covering intake, triage, risk assessment (including suicide risk), safeguarding, and emergency referral.
2. Workforce development. Providers, including counselors and student support officers, will complete a 40-hour competency-based training programme, followed by supervised practice and regular supervision during the first implementation semester. Training will cover structured intake, confidentiality, safeguarding, use of PHQ-9, GAD-7 and WHO-5, suicide and self-harm risk assessment, brief psychoeducation, problem-solving strategies, behavioral activation, anxiety-management techniques, referral documentation, and crisis escalation. OSCE-style assessments will evaluate intake skills, correct use of monitoring tools, risk recognition, brief intervention delivery, care-plan documentation, and appropriate Tier 2 versus Tier 3 referral decisions.
3. Student-facing services. Drop-in hours, scheduled sessions, psychoeducational workshops, and digital self-help resources vetted for cultural and linguistic fit.
4. Data and quality. We will implement a pragmatic monitoring framework to ensure that the service is both effective and safe. This will include indicators of the following: (a) service reach (the proportion of the student population that has contact with the service); (b) access (the median waiting time between first contact and first appointment); (c) Clinical change will be monitored through a predefined minimum outcome set: the Patient Health Questionnaire-9 (PHQ-9) for depressive symptoms, the Generalized Anxiety Disorder-7 (GAD-7) for anxiety symptoms, and the World Health Organization-Five Well-Being Index (WHO-5) for subjective well-being. PHQ-9 and GAD-7 will be administered at intake and at planned review or discharge, while WHO-5 will provide a brief well-being indicator. Portuguese-language versions will be checked for local comprehension and cultural acceptability during piloting. Reliable clinical change will be defined as meaningful pre-post improvement, interpreted alongside attendance, risk status, and student-reported acceptability; (d) safety (the number and nature of serious incidents or adverse events); and (e) acceptability (a single Net Promoter-style rating of satisfaction, complemented by brief qualitative feedback from students). Together, these indicators provide a feasible yet robust basis for continuous quality improvement in routine practice.
5. Referral pathways. We will establish clear and formal agreements (Memoranda of Understanding) with local health and educational services to ensure that students who require more specialized care can be smoothly referred. A simple, shared referral form will be used by all partners, and we will create feedback mechanisms to keep the campus service informed when a student attends an external appointment. This will help reduce the risk of students “getting lost” during the transition between services.

Project documents specify these elements as deliverables for HEIs, the training program, and the monitoring framework; this Study Protocol consolidates them as a coherent implementation pathway.

### Procedural transparency and reproducibility

To support transparency and reproducibility across sites, the protocol specifies a shared operational core while allowing contextual adaptation at each participating HEI. This shared core includes campus-level standard operating procedures for intake, triage, risk assessment, safeguarding, and emergency referral; structured training modules, skills checklists, and supervision ladders; defined student-facing service components; formal referral pathways; and a pragmatic minimum monitoring framework to guide Plan-Do-Study-Act refinement. In practice, this allows institutions to adapt language, staffing, timetables, and infrastructure without losing the common procedural backbone required for cross-site learning and service quality.

### Context: epidemiology and health-system capacity in Angola

The Global Burden of Disease 2021 synthesis in 204 countries/territories showed Central sub-Saharan Africa with the highest age-standardized incidence rate (ASIR) for mental disorders (≈ 8,706 per 100,000 person-years in 2021), and specifically listed Angola among countries with the highest ASIR in 2021. This supports prioritizing scalable, early-access interventions in health education information (HEIs) to reduce the demand on overstretched secondary/tertiary care (The Lancet Psychiatry, 2024).

Workforce and services. Angola’s latest World Health Organization (WHO) Member State mental health profile (2017) reported approximately 0.06 psychiatrists and 0.18 psychologists per 100,000 people; mental health nurses, ≈0.66 per 100,000 people; and a total mental health workforce of ≈1.49 per 100,000 people. Inpatient capacity existed but with minimal dedicated community outpatient infrastructure, highlighting the need to expand first-line, non-specialist services ( World Health Organization, 2017).

Suicide baseline. The most recent World Health Organization (WHO) country-resolved baseline ( WHO, 2021) indicates a suicide mortality rate for Angola in the mid-single-digit range per 100,000, consistent with the World Bank-compiled series (≈ 6.1/100,000 in 2019). Although suicide is multifactorial, campus-level prevention (gatekeeper training, crisis pathways, and postvention) forms a necessary safety net ( World Bank, 2021).

Policy environment. The World Health Organization’s (WHO) 2024 Angola report documents continuing reforms (basic health laws, information systems, and national plans), creating a window for institutionalizing campus services and embedding standardized indicators into university administrative systems ( World Health Organization, 2024).

### Early implementation: evaluation and learning plan

Although this Study Protocol does not present primary outcomes or human participant data, the implementation phase will include the prospective collection of de-identified routine service and implementation data across participating HEIs. These data will cover referral pathways, presenting concerns, waiting times, attendance, number of sessions, level of care, and discharge status. Where feasible, brief validated measures of symptoms or functioning will also be collected over time to capture early clinical change. Additional information on serious incidents, safeguarding events, supervision, fidelity, and student satisfaction will be used to support quality assurance. Data will be reviewed at regular intervals aligned with academic terms and analysed using pragmatic service indicators, early change trajectories, equity-stratified dashboards, and explicit checks on missingness and data quality to inform iterative Plan-Do-Study-Act refinement.

ANGOSAP applies a pragmatic implementation-research approach to support rapid learning while services are being established and scaled across participating higher education institutions (HEIs). The evaluation is designed to be minimally burdensome for clinicians and students while producing actionable evidence on (i) reach and access, (ii) early clinical change, (iii) quality and safety, and (iv) equity in service delivery and outcomes. Data are reviewed at regular intervals aligned with academic terms/semesters, and findings are used to refine pathways, workforce practices, and student-facing communication through iterative Plan-Do-Study-Act (PDSA) cycles.

### Core evaluation features include:

Cohort-style service analytics (routine outcome and flow monitoring): ANGOSAP will maintain de-identified, term-based service panels capturing referral source, presenting concerns (high-level categories), wait times, initial attendance, number of sessions, step/level of care, and discharge status. Where feasible, session-by-session monitoring using brief validated measures will be used to quantify early changes and support measurement-based care. Analyses will prioritize interpretable indicators for leaders (e.g., median wait time, first-appointment uptake, proportion completing an agreed care plan, early change trajectories), and will explicitly document missingness and data-quality checks to avoid misleading inferences. To address variation in digital infrastructure across sites, each institution will use a shared minimum dataset with standardized variable definitions and de-identified student codes. Data will be entered into a secure digital template where feasible; when internet access or equipment is limited, paper forms will be completed locally and transferred to the same template during scheduled data-entry sessions. Each term, sites will report the proportion of missing values for core fields, reasons for missingness when known, duplicate records, inconsistent dates, and incomplete outcome measures. These checks will be reviewed in cross-site supervision meetings before indicators are interpreted.

Quality and safety monitoring: A structured protocol will guide the review of serious incidents and safeguarding events, including documentation of actions taken, referral/escalation pathways, supervision input, and system-level lessons learned. Quality assurance will include periodic fidelity checks on brief interventions (e.g., adherence to core components, appropriate step/triage decisions) and routine supervision records to ensure consistent risk assessment and continuity of care. Student experience will be integrated through lightweight feedback loops (e.g., short satisfaction and acceptability prompts), enabling timely adjustments to appointment processes, confidentiality communication, and service accessibility.

Equity dashboards (stratified performance and outcome review): To ensure that service benefits are distributed fairly, ANGOSAP will implement dashboards that report key indicators stratified by characteristics relevant to inequity in HEI settings (e.g., gender, year of study, disability status, and first-generation status where available and appropriate). These dashboards will be used to detect disparities in reach (who accesses services), access (wait times and uptake), and early outcomes, and to trigger targeted outreach or pathway modifications (e.g., adapted communication, proactive links with student support offices, or tailored referral routes).

Learning network (cross-site implementation learning): A structured learning network will connect HEIs through scheduled case discussions, supervisor forums, and implementation review meetings. These sessions will focus on harmonizing clinical practice, refining stepped-care pathways, and solving operational constraints (e.g., staffing, space, demand surges, and referral coordination). Cross-site learning will also support the standardization of minimum data elements and shared definitions for key indicators, improving comparability while allowing contextual adaptation.

Overall, the intent is to generate usable, decision-oriented evidence for HEI leadership and relevant national stakeholders (including the Ministry of Health), complementing national indicators and supporting system strengthening through integrated, people-centered service models consistent with World Health Organization (WHO) guidance ( World Health Organization, 2024).

### Ethical and consent procedures within implementation

Although no primary outcomes or human participant data are reported in this Study Protocol, the planned implementation model anticipates future participant-facing service activity within participating HEIs. For those activities, informed consent, confidentiality, and data protection procedures will be applied in a way that is proportionate to local regulations, the service setting, and the sensitivity of students’ mental health information. These safeguards are intended to be embedded within intake, documentation, supervision, and referral processes so that implementation remains both feasible and ethically robust across sites.

## Discussion

ANGOSAP’s model is intentionally modest and scalable: it does not replace specialist care; rather, it builds a functional front door for student-relevant concerns, such as stress, anxiety, depressive symptoms, academic scaffolding, and adjustment, while ensuring safe escalation for higher-acuity cases. In the Angolan context of low specialist density and evolving policy frameworks, several aspects are pivotal. Within the Erasmus+ Capacity Building (CBHE) logic, the project treats student mental health not as a “one-off service,” but as a capacity-building package that universities can own and sustain: trained non-specialist providers, a supervision backbone, and a simple data system that helps leadership make decisions without losing sight of students’ lived experience. First, task-sharing is only safe and scalable when supervision is real–regular, structured, and feasible with limited specialist time. Digital and tele-supervision models (including measurement-based peer supervision) provide a practical way to protect quality, support providers, and document competence as services expand across campuses and semesters ( Singla et al., 2024; Poudyal et al., 2024). However, implementation in Angola will face logistical constraints, particularly unstable internet connectivity, uneven access to devices, and the high cost of mobile data in some provinces. To reduce dependence on continuous connectivity, tele-supervision will combine scheduled low-bandwidth video meetings with telephone supervision, asynchronous case summaries, offline documentation templates, and periodic in-person supervision when feasible. These adaptations will be monitored as implementation barriers in the cross-site learning network. Second, ANGOSAP’s monitoring focuses on data that matter to deans, quality leads, and students: reach, waiting times, retention, and reliable change, reported termly and discussed in governance forums. Pragmatic implementation measures can also surface what is blocking delivery (e.g., feasibility, acceptability, and contextual barriers), making improvement actions more targeted and politically actionable ( Yang et al., 2024). This is especially urgent given the documented global burden and increasing trends for common mental disorders ( GBD 2019 Mental Disorders Collaborators, 2019; GBD 2021 Diseases and Injuries Collaborators, 2024; Fan et al., 2025). Third, because trust is everything in university settings, language and culture are implementation strategies: bilingual materials (Portuguese plus locally relevant languages where feasible), culturally grounded framing of distress and help-seeking, and student co-design to avoid “imported” solutions that feel alien or unsafe ( Fischer et al., 2024; Leung et al., 2025). Even where evidence gaps remain (e.g., campus-level prevalence and treatment coverage), the project approach is to build services now–carefully–and learn fast through termly feedback loops that continuously improve equity, safety, and outcomes.

ANGOSAP supports the UN Sustainable Development Goals by establishing university-embedded Psychological and Psychoeducational Support Services in Angola: it advances SDG 3 (Good Health and Well-being) by expanding mental health literacy, early identification, brief evidence-based interventions, and clear referral pathways through a stepped-care model; it contributes to SDG 4 (Quality Education) by strengthening student well-being and learning outcomes via on-campus support services and workforce development; and it promotes SDG 10 (Reduced Inequalities) by embedding equity and inclusion through active outreach and accessible, confidential entry points so that support reaches underserved student groups ( Fan et al., 2025).

### Study status and scope of the protocol

At the time of this Study Protocol, ANGOSAP is in its early implementation phase. The manuscript synthesizes project design and publicly available statistics, and it does not report primary outcomes or human participant data. Several Angola-specific indicators (e.g., 2021–2024 workforce updates and campus-level coverage) will require new data collection and/or improved national reporting pipelines as implementation progresses.

## Ethics and consent

Ethical approval and consent were not required for this Study Protocol because it synthesizes project design and publicly available statistics and does not report primary outcomes or human participant data. No ethics board name or approval reference number is therefore reported at this stage. As outlined in the service model, any future implementation activities involving student-level data will be conducted in accordance with informed consent, confidentiality, and data protection procedures proportionate to local regulations and to the sensitivity of students’ mental health information.

## Data Availability

No new datasets were generated or analysed in support of this Study Protocol. The manuscript presents the rationale, design, and early implementation context of the ANGOSAP project and does not report primary outcomes or human participant data. Accordingly, no study-specific dataset is available for deposition in a repository at this stage. All statistics reported in the manuscript were obtained from publicly available sources cited in the reference list. Not applicable. This Study Protocol describes an Erasmus+ Capacity Building project and does not report a study design with a specific established reporting guideline checklist.
