# Supplementary material for: Task-Sharing and Stepped-Care Mental Health Services in Angolan Higher Education: A Multi-Site Implementation Protocol (ANGOSAP)
Source: Open Res Eur. 2026 Sep 21;6:154. Originally published 2026 May 8. [Version 3] doi: 10.12688/openreseurope.23442.3 (PMC13560541; doi:10.12688/openreseurope.23442.3)
Supplement: Peer review history

# Reviewer report: Reviewer response for version 3

**DOI:** 10.21956/openreseurope.27148.r81497

**Published:** 2026-09-30

**Authors:** Hengki Yandri

**Affiliations:** 1 Institut Agama Islam Negeri Kerinci, Jambi, Jambi, Indonesia

I have carefully reviewed the revised manuscript and the author's responses to my previous comments. I confirm that I have no further comments or revisions to suggest, and I consider the manuscript acceptable in its current form.

Is the study design appropriate for the research question?

Yes

Are sufficient details of the methods provided to allow replication by others?

Yes

Is the rationale for, and objectives of, the study clearly described?

Yes

Are the datasets clearly presented in a useable and accessible format?

Partly

Reviewer Expertise:

Spiritual Counseling; Counseling; Educational Psychology; Mental Health

I confirm that I have read this submission and believe that I have an appropriate level of expertise to confirm that it is of an acceptable scientific standard.

# Reviewer report: Reviewer response for version 2

**DOI:** 10.21956/openreseurope.26110.r79383

**Published:** 2026-08-20

**Authors:** Hengki Yandri

**Affiliations:** 1 Institut Agama Islam Negeri Kerinci, Jambi, Jambi, Indonesia

**Abstract**

 Consider briefly mentioning the number of participating institutions in the abstract to convey scale

 The phrase "validated symptom or functioning measures" could be more specific given that PHQ-9, GAD-7, and WHO-5 are specified in the methods

**Introduction**

*Student-Specific Rationale*

 While the authors have cited macro-level data, the introduction could benefit from stronger justification for the university setting specifically. Consider adding: 1) Brief mention of university student mental health stressors in Angola (e.g., economic pressure, academic transitions, post-conflict generational factors); 2) Evidence on the developmental period of emerging adulthood as a critical window for mental health intervention

*COVID-19 Impact*

 The mention of COVID-19's impact on mental health is appropriate but could be expanded to note how the pandemic specifically affected university students (disrupted education, isolation, economic hardship).

*Terminology Consistency*

 The use of "Health Education Information (HEIs)" appears once (page 6) when "Higher Education Institutions" is used elsewhere. Consider standardizing to HEI throughout.

**Methods and Study Design**

*Measurement Timing*

 The protocol specifies that PHQ-9 and GAD-7 will be administered "at intake and at planned review or discharge." Consider adding: 1) Whether there is a minimum interval between administrations; 2) Whether measurement-based care will be used to guide treatment decisions during the intervention period; 3) A note on what constitutes "meaningful pre-post improvement" (e.g., reliable change index, clinically significant change threshold)

*Training Specificity*

 The OSCE-style assessment is mentioned but could be strengthened with: 1) A brief description of the assessment rubric (e.g., pass/fail criteria, number of stations, standardized patient use); 2) The timeframe for demonstrating competence post-training

*Cultural Adaptation of Measures*

 The authors note that Portuguese versions will be checked for "local comprehension and cultural acceptability." Consider adding: 1) The specific process for this adaptation (e.g., cognitive interviewing, focus groups, back-translation); 2) Whether any modifications to the instruments are anticipated

*Risk Assessment Protocol*

  The protocol appropriately identifies suicide risk assessment as a core component. Consider adding: 1) The specific risk assessment protocol or tool to be used (beyond PHQ-9 item 9); 2) How risk will be documented and communicated

**Results**

 The section is brief and could be strengthened by more explicitly connecting the described outputs to the study aims

 Consider using subheadings to improve structure

**Discussion**

*Generalizability Limitations*

 The discussion would benefit from explicit acknowledgment that: 1) This is a capacity-building project in a specific cultural context; findings may not fully generalize to other African countries; 2) The participating HEIs represent a self-selected group of motivated institutions

*Sustainability Planning*

 While sustainability is briefly mentioned in the author responses, the main discussion could briefly address: 1) The transition plan post-Erasmus+ funding; 2) How institutional buy-in will be maintained; 3) Whether cost-effectiveness will be evaluated

*Stigma and Help-Seeking*

 The protocol mentions stigma but could more explicitly discuss: 1) Whether there are specific cultural conceptualizations of distress in Angola that might affect help-seeking; 2) How the project addresses stigma in its outreach and psychoeducation strategies

*Comparison with Existing Models*

 Consider briefly comparing the ANGOSAP model to other university-based mental health initiatives in sub-Saharan Africa to highlight its unique contributions.

**Conclusion**

 Consider adding a sentence on the potential broader implications for mental health system strengthening in Angola beyond the university setting

Is the study design appropriate for the research question?

Yes

Are sufficient details of the methods provided to allow replication by others?

Yes

Is the rationale for, and objectives of, the study clearly described?

Yes

Are the datasets clearly presented in a useable and accessible format?

Partly

Reviewer Expertise:

Spiritual Counseling; Counseling; Educational Psychology; Mental Health

I confirm that I have read this submission and believe that I have an appropriate level of expertise to confirm that it is of an acceptable scientific standard, however I have significant reservations, as outlined above.

# Response

**Published:** 2026-08-22

**Authors:** Jose Navarro

**Affiliations:** Psychology, Universidad de Cadiz, Cádiz, Andalusia, Spain

COMMENT BY AUTHORS (DR. JOSE I. NAVARRO) AUGUST 2026

 We sincerely thank Dr. Hengki Yandri for the careful and constructive evaluation of our manuscript, ***“Task-Sharing and Stepped-Care Mental Health Services in Angolan Higher Education: A Multi-Site Implementation Protocol (ANGOSAP)”*** ***.*** We appreciate the reviewer’s detailed reading and agree that several suggestions can improve the clarity, reproducibility, and practical value of the protocol. We have revised the manuscript accordingly and respond to each point below.

**Comment 1** Consider briefly mentioning the number of participating institutions in the abstract to convey scale.

**Response:** We agree. The abstract should make the scale of the implementation clearer. We have revised the abstract to specify that ANGOSAP will be implemented across four Angolan higher education institutions, in partnership with European institutions. This helps readers understand the multi-site nature of the protocol from the outset. Proposed revision: ANGOSAP is an Erasmus+-funded capacity-building initiative designed to establish sustainable, university-embedded Psychological and Psychoeducational Support Services across four Angolan higher education institutions.

**Comment 2** **The phrase “validated symptom or functioning measures” could be more specific given that PHQ-9, GAD-7, and WHO-5 are specified in the methods.**

**Response:** We agree. To improve consistency between the abstract and methods, we have replaced the general phrase with the specific instruments used in the protocol: **PHQ-9, GAD-7, and WHO-5**. **Proposed revision:** Early clinical change will be monitored using PHQ-9, GAD-7, and WHO-5, alongside indicators of reach, access, safety, acceptability, and equity. ***Introduction*** Comment 3 **The introduction could benefit from stronger justification for the university setting specifically, including student stressors in Angola and emerging adulthood as a critical window for intervention.** **Response:** We agree that the introduction relied more heavily on macro-level health-system data than on the specific rationale for intervening in universities. We have added a paragraph explaining why higher education institutions are a strategic setting for early mental health support. The revision highlights academic transitions, economic pressure, first-generation university attendance, family expectations, and the broader post-conflict social context in Angola. We also now refer to emerging adulthood as a developmental period in which many mental health problems first appear or consolidate, making early university-based intervention particularly relevant.

**​ Proposed revision:** Universities represent a critical setting for early mental health intervention because students are often navigating academic transition, economic pressure, separation from family support, uncertainty about employment, and, in Angola, the broader effects of post-conflict social and intergenerational adversity. Many students are also first-generation entrants into higher education, which may increase both opportunity and psychosocial strain. Emerging adulthood is a key developmental window in which common mental health problems may first emerge or become persistent; therefore, campus-based services can provide timely, low-stigma, and developmentally appropriate support. Comment 4 **The COVID-19 impact could be expanded to note how the pandemic specifically affected university students.**

**Response:** We agree. We have expanded the COVID-19 discussion to explain its relevance to university students, including disruption of teaching, social isolation, economic hardship, delayed academic progression, and increased stress during educational transitions.

**Proposed revision:** For university students, the COVID-19 pandemic also disrupted face-to-face teaching, reduced peer and institutional support, increased social isolation, and intensified economic hardship for families and students. These factors are especially relevant in higher education systems where digital access and student support structures may be uneven.  

 Comment 5 **“Health Education Information (HEIs)” appears once when “Higher Education Institutions” is used elsewhere. Standardize terminology.**

**Response:** We thank the reviewer for identifying this inconsistency. This was a typographical error. We have corrected the term to **Higher Education Institutions (HEIs)** throughout the manuscript. **Proposed revision:** Higher Education Institutions (HEIs); ( ***Health*** **Education Information** **, [**without using an acronym **])**

***Methods and Study Design***

 Comment 6 **Measurement timing: clarify minimum interval between administrations, whether measurement-based care will guide treatment, and what constitutes meaningful improvement.**

**Response:** We agree. A protocol should specify not only which measures are used but also when and how they inform care. We have revised the methods to state that PHQ-9, GAD-7 and WHO-5 will be administered at intake and again at planned review or discharge, preferably after a minimum interval of approximately four weeks, unless earlier review is clinically indicated. We also clarify that scores will support measurement-based care, helping providers review progress, identify deterioration, and consider escalation. Finally, we define meaningful improvement pragmatically, using reliable change or clinically meaningful score reduction where appropriate, while avoiding overinterpretation as a diagnostic outcome.

**Proposed revision:** PHQ-9, GAD-7 and WHO-5 will be administered at intake and at planned review or discharge, preferably after a minimum interval of four weeks unless earlier reassessment is clinically indicated. Scores will be used as part of measurement-based care to guide review, supervision, and escalation decisions. Meaningful pre-post improvement will be defined using reliable change where feasible and pragmatic clinical thresholds, interpreted alongside attendance, risk status, functional impairment, and student-reported acceptability.

 Comment 7 **Training specificity: strengthen OSCE-style assessment with rubric, pass/fail criteria, number of stations, standardized patient use, and timeframe for competence.**

**Response:** We agree that this detail improves reproducibility. We have expanded the workforce-development section to specify that the OSCE-style assessment will include structured stations using simulated or standardized student scenarios. We also clarify that the assessment will focus on intake, risk identification, use of PHQ-9/GAD-7/WHO-5, brief intervention delivery, care planning, documentation, and Tier 2 versus Tier 3 decision-making. We have added that trainees must demonstrate competence before independent student-facing work.

**Proposed revision:** Competence will be assessed through skills checklists and OSCE-style stations using simulated student scenarios. Stations will assess structured intake, correct use and interpretation of PHQ-9, GAD-7 and WHO-5, recognition of suicide or safeguarding risk, delivery of a brief intervention component, documentation of a care plan, and appropriate Tier 2 versus Tier 3 referral decisions. Providers must meet predefined pass criteria and complete any required supervised practice before delivering student-facing care independently.

 Comment 8 **Cultural adaptation of measures: specify process and whether modifications are anticipated.**

**Response:** We agree. Because the manuscript is a study protocol, we should not claim that the instruments are already culturally validated for Angolan university students unless such validation has been completed. We have therefore added a cautious and scientifically appropriate description of the adaptation process. Portuguese-language versions will be checked through local comprehension review, cognitive interviewing or focus groups with students and staff, and review by local professionals. We do not anticipate changing the scored items unless serious comprehension problems emerge; if changes are required, they will be documented and treated as implementation adaptations rather than assumed psychometric equivalence.

**Proposed revision:** Portuguese-language versions of PHQ-9, GAD-7 and WHO-5 will undergo local comprehension and acceptability checks during piloting, including review by local professionals and cognitive interviews or focus groups with students where feasible. No changes to scored items are anticipated unless substantial comprehension problems are identified. Any adaptation will be documented transparently and interpreted as part of implementation learning rather than as evidence of formal local psychometric validation.

 Comment 9 **Risk assessment protocol: specify risk tool beyond PHQ-9 item 9 and how risk will be documented and communicated.**

**Response:** We agree. PHQ-9 item 9 is useful as an initial safety signal but is not sufficient as a complete suicide risk assessment. We have revised the protocol to include a structured suicide and self-harm risk assessment procedure, including use of a brief risk-assessment tool or structured checklist, documentation of risk level, protective factors, actions taken, supervision review, and referral or emergency escalation where indicated.

**Proposed revision: Any indication of suicidal ideation or self-harm, including a positive response to PHQ-9 item 9, will trigger a same-day structured risk assessment using a brief suicide and self-harm risk protocol. Risk documentation will include current ideation, intent, plan, past self-harm, protective factors, risk level, action taken, supervision input, and referral decision. High or imminent risk will require urgent escalation according to campus emergency procedures and local referral pathways.**

***Results***

 Comment 10 **The Results section is brief and could be strengthened by more explicitly connecting the described outputs to the study aims. Consider using subheadings.**

**Response:** We agree that the Results section can be made clearer. However, because this is a **Study Protocol**, we cannot report clinical or implementation outcomes that have not yet been collected. We have therefore retained a concise Results section but revised it to better organize protocol outputs and connect them to the study aims. We have added subheadings such as **Service implementation outputs**, **Monitoring and quality outputs**, and **Cross-site learning outputs**.

**Proposed revision:** The Results section has been reorganized using subheadings to clarify how the preliminary outputs relate to the protocol aims: service establishment, workforce development, monitoring and quality assurance, and cross-site learning. No primary outcome data are reported at this stage because the manuscript is a protocol.

**Scientific justification:** It would be inappropriate to add empirical findings that are not yet available. In a study protocol, the Results section should describe planned or preliminary implementation outputs only, without implying effectiveness. Any indication of suicidal ideation or self-harm, including a positive response to PHQ-9 item 9, will trigger a same-day structured risk assessment using a brief suicide and self-harm risk protocol. Risk documentation will include current ideation, intent, plan, past self-harm, protective factors, risk level, action taken, supervision input, and referral decision. High or imminent risk will require urgent escalation according to campus emergency procedures and local referral pathways.

***Discussion*** Comment 11 **Generalizability limitations: acknowledge that this is a specific capacity-building project and that participating HEIs are self-selected.**

**Response:** We agree. We have added a limitation noting that ANGOSAP is implemented within a specific cultural, institutional, and funding context. We also acknowledge that participating HEIs are a motivated group of partner institutions and may not represent all Angolan or African universities.

**Proposed revision:** The findings generated by ANGOSAP should be interpreted within the context of a capacity-building project implemented in selected Angolan higher education institutions. Participating institutions are self-selected partners with a high level of motivation and international collaboration experience, which may limit generalizability to other universities in Angola or other African countries.

 Comment 12 **Sustainability planning: address post-Erasmus+ transition, institutional buy-in, and cost-effectiveness evaluation.**

**Response:** We agree that sustainability is central to implementation. We have added a paragraph explaining that the project aims to embed procedures, training capacity, supervision routines, and monitoring systems within participating institutions before the end of Erasmus+ funding. We also clarify that institutional buy-in will be supported through leadership engagement and routine reporting. Regarding cost-effectiveness, we do not present a formal economic evaluation in this protocol because that is beyond the current design; however, we now state that resource use and service activity indicators will be documented to inform future sustainability and cost analyses.

**Proposed revision:** Sustainability will depend on embedding the service model within institutional structures before the end of Erasmus+ funding. The project will therefore prioritize local ownership, leadership engagement, training of campus-based providers, supervision routines, and routine monitoring reports that demonstrate service demand and value. Although a formal cost-effectiveness analysis is beyond the scope of this protocol, service activity, staffing, supervision, and referral indicators will be documented to inform future sustainability and economic evaluation.

 Comment 13 **Stigma and help-seeking: discuss cultural conceptualizations of distress and outreach/psychoeducation strategies.**

**Response:** We agree. We have expanded the discussion of stigma and help-seeking. Because this is a protocol, we avoid making strong empirical claims about cultural conceptualizations of distress without project data. Instead, we state that cultural understandings of distress, stigma, family expectations, and concerns about confidentiality may affect help-seeking, and that the project will address this through psychoeducation, student-facing outreach, confidentiality messaging, and culturally sensitive materials.

**Proposed revision:** Help-seeking may be influenced by stigma, concerns about confidentiality, family expectations, and culturally shaped understandings of psychological distress. ANGOSAP will address these barriers through psychoeducation, student-facing communication, confidentiality messaging, and outreach activities that normalize early support for stress, academic difficulties, anxiety, depressive symptoms, and adjustment problems. Local feedback will be used to refine language and examples so that materials are culturally understandable and acceptable.

 Comment 14 **Comparison with existing models: briefly compare ANGOSAP with other university-based mental health initiatives in sub-Saharan Africa.**

**Response:** We agree that a brief comparison would help position the contribution of ANGOSAP. We have added a short discussion noting that, like other emerging initiatives in sub-Saharan Africa, ANGOSAP uses task-sharing and low-intensity support. Its distinctive contribution is the combination of a multi-site university implementation protocol, stepped care, competency-based workforce development, routine outcome monitoring, and cross-site learning in the Angolan higher education context.

**Proposed revision:** ANGOSAP is aligned with broader efforts in sub-Saharan Africa to expand mental health support through task-sharing, psychoeducation, and low-intensity interventions. Its specific contribution is to adapt these principles to Angolan higher education through a multi-site, university-embedded protocol that combines stepped care, competency-based training, routine monitoring, referral pathways, and cross-site learning.

 Conclusion Comment 15 **Add a sentence on broader implications for mental health system strengthening in Angola beyond the university setting.**

**Response:** We agree. We have added a concluding sentence explaining that, if feasible and acceptable, ANGOSAP may generate practice-based evidence relevant to broader mental health system strengthening in Angola, especially around early identification, task-sharing, referral coordination, and routine monitoring.

**Proposed revision:** Beyond the university setting, the practice-based evidence generated by ANGOSAP may inform wider mental health system strengthening in Angola by demonstrating how early identification, task-sharing, referral coordination, and routine monitoring can be organized in low-resource institutional settings.     We are grateful to Dr. Yandri for comments that have helped us strengthen the manuscript. The revisions improve the specificity of the abstract, clarify the university-specific rationale, strengthen the reproducibility of the measurement and training procedures, provide a more explicit risk-assessment and data-quality framework, and make the discussion more balanced regarding sustainability, stigma, generalizability, and implementation barriers. Where we did not add empirical claims, this was intentional and scientifically appropriate because the manuscript is a Study Protocol and should avoid presenting effectiveness, cultural validation, or cost-effectiveness findings before implementation data are available. I have submitted a new version of the manuscript to the platform, with all changes highlighted in color.

# Reviewer report: Reviewer response for version 2

**DOI:** 10.21956/openreseurope.26110.r76043

**Published:** 2026-06-23

**Authors:** Juan Silvio Cabrera Albert

**Affiliations:** 1 Instituto Superior Politécnico, Lubango, Angola

Dear Dr. Navarro and Dr. Alvaro 

 Thank you very much for the thorough response to the review comments.

 I would like to congratulate you and your co-authors for the professionalism, rigor, and high academic standard reflected in your replies and in the revisions made to the manuscript. It is evident that considerable effort was devoted to carefully addressing the recommendations, which has further strengthened an already valuable contribution.

 I particularly appreciate the constructive and respectful spirit with which the review process was approached. It has been a pleasure to contribute to the evaluation of this important work, and I am confident that the manuscript will be of significant interest to researchers, practitioners, and policymakers working in mental health and higher education.

 My best wishes for the successful continuation of the ANGOSAP project and for your future research endeavors.

 Kind regards,

 Juan Silvio Cabrera Albert

Is the study design appropriate for the research question?

Yes

Are sufficient details of the methods provided to allow replication by others?

Yes

Is the rationale for, and objectives of, the study clearly described?

Yes

Are the datasets clearly presented in a useable and accessible format?

Yes

Reviewer Expertise:

Educational Psychology

I confirm that I have read this submission and believe that I have an appropriate level of expertise to confirm that it is of an acceptable scientific standard.

# Reviewer report: Reviewer response for version 1

**DOI:** 10.21956/openreseurope.25367.r74065

**Published:** 2026-06-08

**Authors:** Juan Silvio Cabrera Albert

**Affiliations:** 1 Instituto Superior Politécnico, Lubango, Angola

This Study Protocol presents the rationale, design, and early implementation framework of the ANGOSAP project, an Erasmus+-funded initiative aimed at establishing university-based Psychological and Psycho-educational Support Services across higher education institutions in Angola. The Protocol addresses a highly relevant and timely issue, namely the growing need for accessible mental health and psycho-educational support services within higher education settings in a context characterized by limited specialist mental health resources and increasing demand for care.

 The manuscript provides a clear overview of the epidemiological and systemic factors that justify the implementation of such services. The authors successfully connect global and national mental health trends, workforce limitations, and higher education needs to justify the development of a stepped-care, task-sharing model adapted to the Angolan context. The Protocol is particularly valuable because it describes an initiative that seeks not only to improve student well-being but also to strengthen institutional capacity and create sustainable structures for mental health support within universities.

 The rationale for the study and its objectives are clearly described. The manuscript effectively explains the challenges facing mental health service provision in Angola and articulates how the ANGOSAP project seeks to respond to these challenges. The objectives are coherent, well-defined, and aligned with the proposed implementation strategy. The justification for adopting a university-based model, emphasizing early intervention, psycho-education, referral pathways, and capacity building, is convincing and supported by relevant literature and policy documents.

 The study design is appropriate for the stated aims. The use of a multi-site, co-designed, stepped-care, and task-sharing framework is consistent with current international recommendations for mental health service development in low-resource settings. The emphasis on local adaptation while maintaining a common operational framework is a notable strength. The Protocol appropriately combines service implementation, workforce development, quality assurance, and continuous evaluation, creating a comprehensive approach that is both pragmatic and potentially scalable.

 The methods are described with sufficient detail to allow replication by other institutions interested in implementing similar services. The manuscript clearly outlines the core design principles, governance procedures, workforce development strategies, student-facing services, referral mechanisms, and monitoring framework. The inclusion of standard operating procedures, supervision structures, quality assurance processes, and implementation learning cycles contributes to transparency and reproducibility. Furthermore, the description of the evaluation framework provides readers with a clear understanding of how service performance and implementation outcomes will be monitored over time.

 The data-related aspects of the protocol are also presented adequately. Given that this is a Study Protocol and does not yet report primary outcome data, the authors appropriately explain that no study-specific datasets are currently available. The planned collection of de-identified routine service data and implementation indicators is described clearly, and the proposed monitoring framework appears suitable for generating actionable evidence while maintaining feasibility in real-world service settings.

 One of the major strengths of the manuscript is its strong focus on implementation science principles. The incorporation of Plan-Do-Study-Act cycles, equity-oriented dashboards, supervision systems, quality monitoring procedures, and cross-site learning networks demonstrates a thoughtful approach to continuous improvement and sustainability. The protocol also highlights the importance of cultural adaptation, student participation, and contextual relevance, which are essential considerations for successful implementation in diverse higher education environments.

 Although the manuscript is scientifically sound and suitable for indexing in its current form, several minor enhancements could further strengthen its contribution for further articles and future publications.

- First, the inclusion of a visual conceptual framework or logic model summarizing the relationships among inputs, activities, outputs, outcomes, and expected impacts would improve readability and provide a concise overview of the implementation model.
- Second, additional information regarding the anticipated number and characteristics of participating institutions could help readers better understand the scale and diversity of the implementation context.
- Third, the authors may consider providing supplementary materials in future versions, such as examples of intake procedures, referral pathways, supervision tools, or monitoring templates, which would facilitate knowledge transfer and adoption by other institutions.

The manuscript could also benefit from a brief discussion of potential implementation challenges and mitigation strategies. Issues such as workforce shortages, service sustainability, student engagement, stigma, and coordination with external health services are likely to influence implementation success and may provide valuable learning opportunities for readers interested in similar initiatives.

 Finally, as the project progresses, future publications reporting implementation outcomes, service utilization data, and lessons learned from participating institutions will be of considerable value to the fields of higher education, mental health, and implementation science.

 In conclusion, this Protocol describes an important and innovative initiative with strong potential to contribute to the development of sustainable university-based mental health and psycho-educational support services in Angola.

 The rationale is clear, the study design is appropriate, the methods are sufficiently described, and the planned data collection and monitoring procedures are adequately presented. The manuscript represents a meaningful contribution to the literature on mental health service development in low-resource higher education settings and merits indexing.

Is the study design appropriate for the research question?

Yes

Are sufficient details of the methods provided to allow replication by others?

Yes

Is the rationale for, and objectives of, the study clearly described?

Yes

Are the datasets clearly presented in a useable and accessible format?

Yes

Reviewer Expertise:

Educational Psychology

I confirm that I have read this submission and believe that I have an appropriate level of expertise to confirm that it is of an acceptable scientific standard.

# Response

**Published:** 2026-06-08

**Authors:** Jose Navarro

**Affiliations:** Psychology, Universidad de Cadiz, Cádiz, Andalusia, Spain

FROM: AUTHOR TO: REVIEWER

 We would like to sincerely thank the reviewer for the careful reading of our manuscript and for the constructive and insightful comments provided. We greatly appreciate the positive assessment of the scientific quality and relevance of the work. Following these valuable suggestions, we have carefully revised the manuscript and addressed each of the points raised. Below, we provide a detailed response outlining the modifications introduced and clarifications added in order to further strengthen the manuscript and improve its clarity, completeness, and potential usefulness for future research and implementation efforts.

- *Visual conceptual framework or logic model summarizing the relationships among inputs, activities, outputs, outcomes, and expected impacts to provide readers of the article a concise overview of the implementation model.*

**Figure1. Conceptual Framework and Logic Model of the ANGOSAP** **Implementation Model** **Inputs** **Activities** **Outputs** **Short- and Medium-Term Outcomes** **Long-Term Impacts** Erasmus+ funding and institutional support Co-design workshops with HEI stakeholders University-specific service implementation plans Increased awareness and mental health literacy among students Improved student well-being and quality of life Partner universities and governance structures Development of standard operating procedures (intake, triage, risk assessment, safeguarding, referral) Standardized operational protocols across participating HEIs Improved access to psychological and psychoeducational support services Enhanced academic engagement, retention, and success Psychologists, supervisors, and trained staff Competency-based training and supervision programs Trained service providers and supervisors Reduced waiting times and improved service accessibility Stronger student support ecosystems within universities Digital resources and psycho-educational materials Delivery of psychoeducation, workshops, self-help resources, and brief interventions Student-facing services operational across campuses Early identification and management of mental health and learning-related difficulties Reduced burden on overstretched specialist mental health services Partnerships with health and educational services Establishment of referral and safeguarding pathways Formalized referral agreements and collaborative networks Improved continuity of care and referral effectiveness Sustainable and integrated university-based mental health services Monitoring systems and implementation framework Routine data collection, quality assurance, supervision, and PDSA cycles Service dashboards, quality indicators, and implementation reports Improved quality, safety, acceptability, and equity of service delivery Evidence-informed policy and institutional decision-making Equity and inclusion strategy Targeted outreach to vulnerable and underserved student groups Equity-focused monitoring and outreach activities Increased service utilization among underrepresented groups Reduced inequalities in access to mental health and psychoeducational support **Cross-Cutting Implementation Mechanisms** The entire model is supported by four cross-cutting mechanisms that operate across all stages:

1. **Stepped-Care Approach** - ensuring that students receive the least intensive and most appropriate intervention according to their needs.
2. **Task-Sharing and Supervision** - expanding service capacity through trained providers supported by structured supervision.
3. **Continuous Quality Improvement** - using Plan-Do-Study-Act (PDSA) cycles and routine monitoring to refine service delivery.
4. **Equity and Inclusion** - promoting accessible, culturally responsive, and student-centered services for diverse populations.

**Theory of Change** By strengthening institutional capacity, training personnel, establishing structured support pathways, and embedding continuous evaluation mechanisms, ANGOSAP is expected to increase access to timely psychological and psychoeducational support. Improved access and service quality should lead to better mental health, enhanced academic functioning, greater educational inclusion, and ultimately contribute to sustainable mental health system strengthening within Angolan higher education institutions.  

- *Additional information regarding the anticipated number and characteristics of participating institutions to help readers better understand the scale and diversity of the implementation context.*

Participating Institutions and Implementation Context The ANGOSAP consortium brings together a diverse group of higher education institutions (HEIs) across Angola, reflecting variations in geographical location, institutional size, governance structures, academic profiles, and student demographics. Participating institutions include both public and private HEIs located in different regions of the country, thereby enabling the implementation model to be tested across heterogeneous educational environments. The consortium encompasses universities and polytechnic institutions serving students enrolled in a broad range of disciplines, including education, health sciences, engineering, social sciences, economics, law, and humanities. Student populations vary considerably between institutions, ranging from medium-sized campuses with a few thousand students to larger institutions serving several tens of thousands of students. This diversity provides an opportunity to assess the feasibility, acceptability, and adaptability of the ANGOSAP model under different organizational and resource conditions. Participating institutions also differ in their existing student support structures and mental health resources. While some institutions have limited access to psychological support services, others possess emerging counseling or student welfare units that can serve as foundations for the development of more comprehensive Psychological and Psychoeducational Support Services. Such variability is expected to generate valuable implementation learning regarding the factors that facilitate or hinder service establishment and sustainability. From a workforce perspective, institutions exhibit different levels of access to qualified psychologists, educational specialists, and student support personnel. The project therefore adopts a flexible implementation strategy based on task-sharing, competency-based training, and structured supervision to ensure that core service components can be delivered consistently despite local variations in human resource capacity. The inclusion of institutions operating in diverse socioeconomic, cultural, and linguistic contexts further strengthens the project's external validity. By testing the implementation framework across multiple settings, ANGOSAP seeks to generate transferable knowledge that may inform the development of university-based mental health and psychoeducational support services both within Angola and in other low- and middle-income countries facing similar challenges. Future reports arising from the project will provide detailed information on the number of participating institutions, student population coverage, service utilization rates, workforce composition, and implementation outcomes, enabling a more comprehensive assessment of scalability and sustainability. **Table 1. Characteristics of participating higher education institutions in the ANGOSAP Consortium** **Institution** **Region/Province** **Type** **Approximate Student Population** **Existing Student Support Resources** **Planned ANGOSAP Service Components**         Student affairs office; limited counseling support Psychoeducation, brief interventions, referral pathways **Abbreviations:** ANGOSAP: Angola Psychological and Psychoeducational Support Services; HEI: Higher Education Institution. **Note:** The table should be completed using institution-specific data obtained during the project mapping and needs-assessment phase. Inclusion of this information would allow readers to better understand the geographical coverage, institutional diversity, implementation context, and scalability potential of the ANGOSAP model.  

- *Provide supplementary materials, such as examples of intake procedures, referral pathways, supervision tools, or monitoring templates, which would facilitate knowledge transfer and adoption by other institutions.*

**Suggested supplementary materials for ANGOSAP implementation** To facilitate knowledge transfer, transparency, and replication, the authors may consider including the following supplementary materials as online appendices: Supplementary Appendix A. Student Intake and Initial Assessment Form A standardized intake template that could include:

- Student demographic information
- Academic program and year of study
- Presenting concerns
- Previous support received
- Risk screening questions
- Consent and confidentiality statements
- Referral source
- Initial triage decision

**Purpose:** To promote consistency in data collection and service access across participating institutions.   Supplementary Appendix B. Triage and Referral Decision Pathway A visual flowchart describing:

1. Initial student contact
2. Screening and risk assessment
3. Allocation to stepped-care level
4. Criteria for brief intervention
5. Criteria for referral to specialized services
6. Emergency and crisis management procedures
7. Follow-up and case closure

**Purpose:** To ensure consistency and safety in decision-making processes.   Supplementary Appendix C. Risk Assessment and Safeguarding Protocol A practical protocol covering:

- Suicide risk assessment procedures
- Self-harm concerns
- Severe psychological distress
- Gender-based violence disclosures
- Safeguarding responsibilities
- Emergency referral procedures
- Documentation requirements

**Purpose:** To strengthen clinical governance and student safety.   Supplementary Appendix D. Supervision Framework and Competency Checklist Examples of:

- Supervision schedules
- Session documentation templates
- Competency assessment rubrics
- Skills observation forms
- Fidelity monitoring tools
- Escalation procedures for complex cases

**Purpose:** To support quality assurance and workforce development.   Supplementary Appendix E. Monitoring and Evaluation Dashboard Sample indicators and reporting templates including: **Domain** **Indicator** Reach Number and proportion of students accessing services Access Median waiting time to first appointment Engagement Attendance rates and number of completed sessions Outcomes Change in symptom or functioning measures Safety Number of serious incidents reported Acceptability Student satisfaction scores Equity Utilization rates by gender, disability status, year of study, and other relevant characteristics **Purpose:** To support routine service monitoring and continuous quality improvement.   Supplementary Appendix F. Plan-Do-Study-Act (PDSA) Improvement Template A standardized form documenting:

- Identified problem
- Improvement action
- Implementation period
- Results achieved
- Lessons learned
- Recommended next steps

**Purpose:** To facilitate implementation learning across institutions.   Supplementary Appendix G. Service Logic Model and Theory of Change A visual framework presenting:

- Inputs
- Activities
- Outputs
- Short-term outcomes
- Intermediate outcomes
- Long-term impacts

**Purpose:** To provide a concise overview of the intervention model and implementation assumptions.   Supplementary Appendix H. Minimum Data Set Dictionary A data dictionary specifying:

- Variable names
- Definitions
- Coding procedures
- Data collection frequency
- Responsible personnel

**Purpose:** To improve standardization, comparability, and data quality across sites.  

- *Potential implementation challenges and mitigation strategies. Issues such as workforce shortages, service sustainability, student engagement, stigma, and coordination with external health services are likely to influence implementation success and may provide valuable learning opportunities for readers interested in similar initiatives.*

Potential implementation challenges and mitigation strategies As with many mental health service innovations implemented in low-resource settings, ANGOSAP is likely to encounter a range of operational, organizational, and contextual challenges during implementation and scale-up. Acknowledging these challenges is important not only for transparency but also for informing future replication efforts in similar higher education contexts. One anticipated challenge relates to **workforce capacity and availability**. Angola continues to experience a limited supply of mental health specialists, particularly outside major urban centers. Variability in the availability of psychologists and other qualified personnel across participating institutions may affect service delivery and supervision capacity. To mitigate this risk, ANGOSAP adopts a task-sharing approach supported by competency-based training, structured supervision, tele-supervision mechanisms, and standardized protocols designed to maintain service quality while optimizing available human resources. A second challenge concerns the **long-term sustainability of services** beyond the Erasmus+ funding period. Institutional priorities, financial constraints, and staff turnover may affect the continuity of support services. Sustainability will therefore depend on the progressive integration of Psychological and Psychoeducational Support Services into existing university structures, budgets, strategic plans, and quality assurance systems. Capacity-building efforts targeting institutional leadership and administrative personnel may further strengthen local ownership and sustainability. **Student engagement and service utilization** also represent important implementation considerations. Despite the availability of services, students may be reluctant to seek support because of limited mental health literacy, concerns about confidentiality, competing academic demands, or lack of awareness regarding available resources. To address these barriers, ANGOSAP incorporates psychoeducational activities, awareness campaigns, student co-design processes, flexible access pathways, and culturally responsive communication strategies intended to increase trust, visibility, and service acceptability. Relatedly, **mental health stigma** remains a potential obstacle to help-seeking behavior. Negative perceptions surrounding mental health difficulties may discourage students from accessing support or disclosing their needs. The project's emphasis on mental health literacy, peer engagement, confidential service provision, and normalization of help-seeking is expected to contribute to stigma reduction over time. Monitoring patterns of service utilization may also help identify groups that remain underserved and require targeted outreach strategies. Another challenge involves **coordination with external health and social services**. Effective stepped-care models depend on reliable referral pathways for students requiring specialized assessment or treatment. However, variations in service availability, referral procedures, communication mechanisms, and geographic accessibility may complicate continuity of care. ANGOSAP seeks to address this issue through formal referral agreements, standardized referral procedures, and the establishment of collaborative relationships with local healthcare providers and relevant community services. The project may also encounter challenges related to **data quality, monitoring, and evaluation**. Routine service data collection can be affected by incomplete records, inconsistent reporting practices, or limited technical infrastructure. To minimize these risks, the project includes a minimum dataset framework, standardized monitoring procedures, staff training, and regular data-quality reviews as part of its continuous improvement strategy. Finally, the diversity of participating institutions may create challenges related to **implementation fidelity and contextual adaptation**. Differences in institutional culture, infrastructure, staffing, and student demographics may require local modifications to service delivery models. ANGOSAP addresses this tension by defining a common operational core while allowing contextual adaptation through co-design processes, thereby balancing standardization with flexibility. Importantly, these challenges should not be viewed solely as barriers but also as opportunities for implementation learning. Systematic documentation of obstacles, adaptations, and solutions throughout the project will contribute valuable evidence for the development of sustainable university-based mental health services in Angola and other low- and middle-income country settings.

# Reviewer report: Reviewer response for version 1

**DOI:** 10.21956/openreseurope.25367.r74061

**Published:** 2026-05-21

**Authors:** Rohman Hikmat

**Affiliations:** 1 Universitas Padjadjaran, Sumedang, Indonesia; 2 Prince of Songkla University (Ringgold ID: 26686), Hat Yai, Songkhla, Thailand

**1. Title**

- **Critique:** The title is overly wordy and administrative ("...a brief report on rationale, design, and early implementation context").
- **Suggestion:** **Revise for impact and clarity.** Focus on the core innovation—the task-sharing model in the Angolan university context. *Example: "Task-Sharing and Stepped-Care Mental Health Services in Angolan Higher Education: A Multi-Site Implementation Protocol (ANGOSAP)."*

**2. Abstract**

- **Critique:** The abstract identifies the problem (workforce shortage) and the solution (ANGOSAP) but lacks a clear description of the *specific* primary metrics that will define "early implementation success".
- **Suggestion:** Include the specific **monitoring indicators** (e.g., reach, wait times, reliable clinical change) directly in the abstract so readers understand the evaluative rigor of the protocol.

**3. Introduction**

- **Critique:** The manuscript does an excellent job of citing macro-level statistics (0.06 psychiatrists per 100,000 population). However, it lacks a **student-specific rationale**. Why is the university setting the optimal entry point in Angola compared to community health centers?
- **Suggestion:** Bolster the introduction with literature on the unique mental health stressors facing **Angolan university students** (e.g., transition, economic pressure, post-conflict generational trauma) to justify the university-embedded focus.

**4. Methods and Study Design**

- **Critique (Major Concern - Replication):** The manuscript mentions "brief, evidence-based psychological interventions" and "validated symptom or functioning scales" but **fails to name them**. A protocol must be reproducible.
- **Requirement:** Specify exactly which tools will be used (e.g., PHQ-9, GAD-7, or WHO-5). Simply stating "validated scales" is insufficient.
- **Critique (Task-Sharing Detail):** You mention using "trained non-specialists" such as counselors and student support officers.
- **Requirement:** Provide more detail on the **competency-based training package**. What is the duration of the training? What are the "OSCE-style assessments" measuring specifically?.
- **Critique (Stepped Care):** The transition between Tier 2 (brief intervention) and Tier 3 (specialist referral) is vague.
- **Requirement:** Define the **triage criteria** or clinical thresholds that trigger an escalation to specialist care.

**5. Data and Quality Monitoring**

- **Critique:** You propose a "pragmatic monitoring framework". However, in a low-resource setting like Angola, data missingness is a significant risk.
- **Suggestion:** Expand on the "explicit checks on missingness and data quality". How will de-identified data be collected across sites with potentially varying digital infrastructure?.

**6. Discussion**

- **Critique:** The discussion is somewhat repetitive of the rationale. It glosses over the **major implementation barriers** specific to Angola.
- **Suggestion:** Critically discuss the **logistical hurdles** of your model. For example, if "tele-supervision" is a core pillar, how will you address unstable internet connectivity or the high cost of data for staff in rural Angolan provinces?. Addressing these challenges will make the protocol more "honest" and valuable to other implementers.

**7. Conclusions**

- **Critique:** The conclusion claims the framework is "feasible". As a protocol for an "early implementation phase," this is a premature claim.
- **Suggestion:** Reframe the conclusion to state that the protocol *aims to test the feasibility* and provide a roadmap for how the "practice-based evidence" generated will be used to influence Angolan national health policy.

Is the study design appropriate for the research question?

Yes

Are sufficient details of the methods provided to allow replication by others?

Partly

Is the rationale for, and objectives of, the study clearly described?

Yes

Are the datasets clearly presented in a useable and accessible format?

No

Reviewer Expertise:

mental health, adolescents, bullying, domestic violence, anxiety, resilience

I confirm that I have read this submission and believe that I have an appropriate level of expertise to confirm that it is of an acceptable scientific standard, however I have significant reservations, as outlined above.

# Response

**Published:** 2026-05-24

**Authors:** Jose Navarro

**Affiliations:** Psychology, Universidad de Cadiz, Cádiz, Andalusia, Spain

**COMMENTS TO THE reviewer – OPEN RESEARCH EUROPE ( *STUDY PROTOCOL*)** *TITLE: Building university-based psychological and psychoeducational services in Angola (ANGOSAP): a brief report on rationale, design, and early implementation context*.   REVIEWER COMMENTS ANSWER FROM AUTHORS/NEW TEXT

**1. Title**

- **Critique:** The title is overly wordy and administrative ("...a brief report on rationale, design, and early implementation context").
- **Suggestion:** **Revise for impact and clarity.** Focus on the core innovation—the task-sharing model in the Angolan university context. *Example: "Task-Sharing and Stepped-Care Mental Health Services in Angolan Higher Education: A Multi-Site Implementation Protocol (ANGOSAP)."*

**New title** *Task-Sharing and Stepped-Care Mental Health Services in Angolan Higher Education: A Multi-Site Implementation Protocol (ANGOSAP)*

**2. Abstract**

- **Critique:** The abstract identifies the problem (workforce shortage) and the solution (ANGOSAP) but lacks a clear description of the *specific* primary metrics that will define "early implementation success".
- **Suggestion:** Include the specific **monitoring indicators** (e.g., reach, wait times, reliable clinical change) directly in the abstract so readers understand the evaluative rigor of the

**(new text in yellow)** **Methods** This Study Protocol describes the rationale, service model, and framework of ANGOSAP. The project uses a co-designed, stepped-care, task-sharing approach adapted to each institution. Core components include campus-level procedures for intake, triage, risk assessment, safeguarding, and referral; competency-based training and supervision; psychoeducation, brief psychological interventions, and digital resources; referral pathways; and pragmatic monitoring.

**Early implementation success will be assessed using predefined indicators: service reach, median waiting time from first contact to first appointment, first-appointment uptake, care-plan completion, reliable clinical change on validated symptom or functioning measures, serious incidents, acceptability, and equity-stratified access and outcomes.** **.**

**Methods and Study Design**

- **Critique (Major Concern - Replication):** The manuscript mentions "brief, evidence-based psychological interventions" and "validated symptom or functioning scales" but **fails to name them**. A protocol must be reproducible.
- **Requirement:** Specify exactly which tools will be used (e.g., PHQ-9, GAD-7, or WHO-5). Simply stating "validated scales" is insufficient.           

- **Critique (Task-Sharing Detail):** You mention using "trained non-specialists" such as counselors and student support officers.
- **Requirement:** Provide more detail on the **competency-based training package**. What is the duration of the training? What are the "OSCE-style assessments" measuring specifically?. 
- **Critique (Stepped Care):** The transition between Tier 2 (brief intervention) and Tier 3 (specialist referral) is vague.
- **Requirement:** Define the **triage criteria** or clinical thresholds that trigger an escalation to specialist care.

 
**Revised text for “Data and quality”** Clinical change will be monitored through a predefined minimum outcome set: the Patient Health Questionnaire-9 (PHQ-9) for depressive symptoms, the Generalized Anxiety Disorder-7 (GAD-7) for anxiety symptoms, and the World Health Organization-Five Well-Being Index (WHO-5) for subjective well-being. PHQ-9 and GAD-7 will be administered at intake and at planned review or discharge, while WHO-5 will provide a brief well-being indicator. Portuguese-language versions will be checked for local comprehension and cultural acceptability during piloting. Reliable clinical change will be defined as meaningful pre-post improvement, interpreted alongside attendance, risk status, and student-reported acceptability.    

**2. Workforce development.** Providers, including counselors and student support officers, will complete a 40-hour competency-based training programme, followed by supervised practice and regular supervision during the first implementation semester. Training will cover structured intake, confidentiality, safeguarding, use of PHQ-9, GAD-7 and WHO-5, suicide and self-harm risk assessment, brief psychoeducation, problem-solving strategies, behavioral activation, anxiety-management techniques, referral documentation, and crisis escalation. OSCE-style assessments will evaluate intake skills, correct use of monitoring tools, risk recognition, brief intervention delivery, care-plan documentation, and appropriate Tier 2 versus Tier 3 referral decisions.  

**b) Stepped care.**

 Tier 1 will include mental health literacy, psychoeducation, digital self-help resources, and low-intensity workshops.

 Tier 2 will provide brief structured interventions, including problem-solving strategies, behavioral activation, anxiety-management techniques, and academic adjustment support, for students with mild-to-moderate symptoms and no immediate safety concerns.

 Tier 3 referral to specialist care will be triggered by active suicidal ideation or self-harm risk, psychotic or manic symptoms, severe substance misuse, safeguarding concerns, severe functional impairment, clinical deterioration during Tier 2, non-response after a brief intervention period, or severe PHQ-9/GAD-7 scores. Any positive response to PHQ-9 item 9 will require same-day risk assessment and, where indicated, urgent referral.

***COMMENTS BY AUTHORS:*** We thank the reviewer for this important observation. We have revised the “Stepped care” subsection to specify the clinical and safety criteria that trigger escalation to specialist care, including suicidal ideation or self-harm risk, psychotic or manic symptoms, severe substance misuse, safeguarding concerns, severe functional impairment, deterioration during Tier 2, non-response to brief intervention, and severe PHQ-9/GAD-7 scores. We also specify that any positive response to PHQ-9 item 9 will require same-day risk assessment and urgent referral when indicated. This revision improves the reproducibility and safety of the stepped-care pathway.****

**Data and Quality Monitoring**

- **Critique:** You propose a "pragmatic monitoring framework". However, in a low-resource setting like Angola, data missingness is a significant risk.
- **Suggestion:** Expand on the "explicit checks on missingness and data quality". How will de-identified data be collected across sites with potentially varying digital infrastructure?.

  To address variation in digital infrastructure across sites, each institution will use a shared minimum dataset with standardized variable definitions and de-identified student codes. Data will be entered into a secure digital template where feasible; when internet access or equipment is limited, paper forms will be completed locally and transferred to the same template during scheduled data-entry sessions. Each term, sites will report the proportion of missing values for core fields, reasons for missingness when known, duplicate records, inconsistent dates, and incomplete outcome measures. These checks will be reviewed in cross-site supervision meetings before indicators are interpreted.  

***COMENTS BY AUTHORS:*** We thank the reviewer for highlighting the risk of data missingness in a low-resource setting. We agree that this required clearer operational detail. We have revised the “Cohort-style service analytics” subsection to specify how de-identified data will be collected across sites with varying digital capacity. The revised text now describes the use of a shared minimum dataset, standardized variable definitions, de-identified student codes, secure digital templates where feasible, and paper-to-digital transfer procedures when digital infrastructure is limited. We also specify termly checks for missing values, duplicate records, inconsistent dates, incomplete outcome measures, and documented reasons for missingness. These procedures are intended to improve data quality while remaining feasible across participating Angolan institutions.****

**Discussion**

- **Critique:** The discussion is somewhat repetitive of the rationale. It glosses over the **major implementation barriers** specific to Angola.
- **Suggestion:** Critically discuss the **logistical hurdles** of your model. For example, if "tele-supervision" is a core pillar, how will you address unstable internet connectivity or the high cost of data for staff in rural Angolan provinces?. Addressing these challenges will make the protocol more "honest" and valuable to other implementers.

    However, implementation in Angola will face logistical constraints, particularly unstable internet connectivity, uneven access to devices, and the high cost of mobile data in some provinces. To reduce dependence on continuous connectivity, tele-supervision will combine scheduled low-bandwidth video meetings with telephone supervision, asynchronous case summaries, offline documentation templates, and periodic in-person supervision when feasible. These adaptations will be monitored as implementation barriers in the cross-site learning network.

***COMMENTS FROM AUTHORS:*** We have revised the Discussion to acknowledge unstable internet connectivity, uneven access to devices, and the cost of mobile data as potential constraints for tele-supervision. We now specify mitigation strategies, including low-bandwidth meetings, telephone supervision, asynchronous case summaries, offline documentation templates, and periodic in-person supervision where feasible. This revision provides a more realistic and transferable account of implementation challenges.    

**Conclusions**

- **Critique:** The conclusion claims the framework is "feasible". As a protocol for an "early implementation phase," this is a premature claim.
- **Suggestion:** Reframe the conclusion to state that the protocol *aims to test the feasibility* and provide a roadmap for how the "practice-based evidence" generated will be used to influence Angolan national health policy.

 ANGOSAP provides a structured protocol to test the feasibility of university-based psychological and psychoeducational support in Angola. By combining capacity building, supervision, referral integration, and routine monitoring, the project will generate practice-based evidence on reach, access, safety, acceptability, and early clinical change. These findings will inform service refinement across participating institutions and provide a roadmap for engaging national stakeholders, including health and higher education authorities, in future policy development.

***COMMENTS FROM AUTHORS:*** We have revised the conclusion to state that ANGOSAP aims to test feasibility rather than claim it. The revised text also clarifies how the practice-based evidence generated through routine monitoring will be used to refine services and inform engagement with Angolan health and higher education stakeholders for future policy development.    
